# Capacity of Heterogeneous Mobile Wireless Networks with *D*-Delay Transmission Strategy

**DOI:** 10.3390/s16040425

**Published:** 2016-03-25

**Authors:** Feng Wu, Jiang Zhu, Zhipeng Xi, Kai Gao

**Affiliations:** School of Electronic Science and Engineering, National University of Defense Technology, Changsha 410073, China; wufeng_paper@outlook.com (F.W.); xzp_paper@163.com (Z.X.); gaokai000@nudt.edu.cn (K.G.)

**Keywords:** network capacity, heterogeneous, mobile networks, delay constraints

## Abstract

This paper investigates the capacity problem of heterogeneous wireless networks in mobility scenarios. A heterogeneous network model which consists of *n* normal nodes and *m* helping nodes is proposed. Moreover, we propose a *D*-delay transmission strategy to ensure that every packet can be delivered to its destination nodes with limited delay. Different from most existing network schemes, our network model has a novel two-tier architecture. The existence of helping nodes greatly improves the network capacity. Four types of mobile networks are studied in this paper: i.i.d. fast mobility model and slow mobility model in two-dimensional space, i.i.d. fast mobility model and slow mobility model in three-dimensional space. Using the virtual channel model, we present an intuitive analysis of the capacity of two-dimensional mobile networks and three-dimensional mobile networks, respectively. Given a delay constraint *D*, we derive the asymptotic expressions for the capacity of the four types of mobile networks. Furthermore, the impact of *D* and *m* to the capacity of the whole network is analyzed. Our findings provide great guidance for the future design of the next generation of networks.

## 1. Introduction

Network capacity is one of the key factors of wireless networks. The first study of network capacity is carried out by Gupta and Kumar in [1]. They investigated the throughput capacity of a wireless network where *n* nodes are randomly distributed, and find that the per-node throughput capacity is $\Theta \left(\sqrt{\frac{W}{n\;\text{log}\;n}}\right)$, where *W* is the highest transmission rate of each node. Their finding implies that as the size of the network goes to infinity, the per-node throughput will decrease to zero. Following this work, extensive studies have been conducted to achieve a tighter capacity bound [2,3,4,5,6,7,8,9,10,11,12,13,14,15].

Some researchers propose to add some base stations to help with long distance transmissions in networks, which are called “hybrid networks” [16,17,18,19,20,21]. Compared with pure *ad hoc* networks, the base stations are neither sources nor destinations, they work only as relay nodes. Liu *et al.* [16] studied the capacity of networks where the base stations are regularly placed and the distribution of normal nodes is independent and identically distributed (i.i.d.). They obtain the asymptotic expressions on aggregate throughput capacity and maximize the capacity under different channel allocation schemes. Kozat *et al.* [17] investigated the capacity bound of the networks where base stations and nodes are both randomly deployed. They prove that the per-node throughput is $\Theta \left(\frac{W}{\text{log}\;n}\right)$when the number of nodes and the number of base stations have the same order. Zemlianov *et al.* [18] considered the case that the nodes are randomly distributed and the base stations are arbitrarily placed. They proved that the network capacity depends on the number of base stations. Zhang *et al.* [19] proposed a network model where delay constraint is considered and each node is equipped with directional antennas. They also analyzed how the number of base stations and the beamwidth of directional antennas affect the network capacity.

There are also some papers focusing on guaranteeing the QoS of some special scenarios over hybrid wireless networks [22,23,24]. The distortion-aware concurrent multipath transfer (CMT-DA) solution, delay stringent coded transmission (ASCOT) and goodput-aware load distribution (GALTON) are proposed in these papers. By evaluating the performance through experiments, the authors prove that the proposed models outperform existing transmission schemes.

A large volume of literature proves that adding base stations is an efficient way to improve the network capacity. However, this has some disadvantages. First, to improve the throughput capacity significantly, a large number of base stations should be set up, which is very expensive. Second, it takes a lot of time to establish such a wired network. Third, in some practical cases such as battlefields and rescue tasks, there is little time to set up base stations.

Therefore, compared with establishing base stations, deploying some powerful helping nodes is an easy and efficient way to improve the network capacity. Networks which consist of normal nodes and helping nodes are called “heterogeneous networks”. Though there are plenty of research studies on heterogeneous networks [25,26,27,28,29], most of them focus on routing protocol design [22,23] and MAC protocol design [27,28], instead of network capacity. One study focused on network capacity [29] investigated the capacity of two types of networks: regular heterogeneous networks and random heterogeneous networks. 

In the literature mentioned above, the nodes are usually assumed to be stable. Some researchers focus on the networks where the nodes are mobile. Grossglauser and Tse for the first time introduced mobility to networks [30]. They proved that the network capacity reaches Θ(*W*) in mobile networks, while the transmission delay will go to infinity in their network model. Inspired by this work, many researchers have engaged in the study of the capacity of mobile networks [30,31,32,33,34,35,36,37,38,39,40]. Of all the mobility models, the i.i.d. model is the simplest and the most commonly used one. Neely *et al.* [31] analyzed the delay-throughput tradeoff of i.i.d. mobility networks. They prove that the throughput capacity is $\Omega \left(\frac{D}{n}\right)$, where *n* is the number of nodes and *D* is the transmission delay. The mobile network in [31] is substantially a fast mobility model. In this model, the data transmissions and the node mobility are assumed to be on the same time scale. Hence there exists only one-hop transmission in a time slot. In [32], Toumpis and Goldsmith considered the case that the node mobility is much slower than the data transmissions. In their network model, in single time slot, packets can be delivered by multi-hop transmissions. They show that the delay-throughput tradeoff is $\Theta \left(\sqrt{\frac{D}{n}}\text{log}\;n\right)$ in their i.i.d. slow mobility network model. Other researchers have also contributed a lot to the studies on the capacity of i.i.d. mobility networks.

The random walk model is also an important mobility model. Grammal *et al.* [33] first introduced the random walk model. Later on, the throughput capacities of per source-destination pair in fast mobility networks [34] and slow mobility networks were investigated [35]. Besides i.i.d. mobility model and random walk model, other mobility models such as Brownian model and hybrid random walk model are also well studies [36,37].

However, most of the literature above focuses on the two-dimensional mobility model or one-dimensional mobility model. In fact, as wireless technologies are rapidly developing, wireless networks are extending from two-dimensional to three-dimensional space. Moreover, in many scenarios, the network can be better modeled by a three-dimensional space instead of a two-dimensional space. For instance, in modern battlefields, a large amount of wireless networks are emerging which consist of lots of military units such as fleets, troops and aircrafts. Hence, we can foresee that studies on three-dimensional networks will be of great significance.

In previous studies, only a few researchers studied the capacity problems in three-dimensional networks [41,42,43]. The three-dimensional arbitrary network model and three-dimensional random network model are, for the first time, proposed in [41]. The authors calculated the transport capacity and throughput capacity of the two types of models, respectively. Li *et al.* [42] proposed a three-dimensional network model where the distributions of nodes are inhomogeneous. They obtained the asymptotic expressions on the throughput capacity of their model. Cai *et al.* [43] considered the three-dimensional networks with UWB technologies. They also obtained the throughput capacity.

In this paper, we consider heterogeneous networks with *n* normal nodes and *m* helping nodes. In our network model, the helping nodes are more powerful than the normal nodes and just work in routing and relaying. They are neither the source nodes nor the destination nodes. Moreover, we adopt the delay constraint *D* in our routing strategy. Concretely, if the source can send the packets to its destination within a time slot *D*, the packets will be transmitted in multi-hop fashion, which is also called *ad hoc* transmission mode. If the transmission delay exceeds *D* time slots, the packets will be delivered to the destination with the help of helping nodes, which is called helping transmission mode. We adopt the i.i.d. mobility model in this paper. Moreover, we consider two time scales: fast mobility and slow mobility. Besides, we also consider two cases, where all the nodes are distributed in two-dimensional space and where all of them are distributed in three-dimensional space. We first give an intuitive analysis under virtual channel systems. Then the asymptotic expressions are obtained by strict proof. Based on the expressions, we consider the impact of *m* and *D* to the per-node throughput capacity and have some interesting findings. Our main contributions can be summarized as follows:

(1) We propose a new heterogeneous mobile network model, which consists of *n* mobile normal nodes and *m* stable helping nodes. The helping nodes are more powerful and they only work in routing and relaying data. Compared with the traditional hybrid network model, setting up such a backbone with helping nodes saves a lot of time and money.
(2) According to the time scales and space dimensions, we consider four types of network model: two-dimensional i.i.d. fast mobility model, two-dimensional i.i.d. slow mobility model, three-dimensional i.i.d. fast mobility model and three-dimensional i.i.d. slow mobility model. We derive the capacities of the four types of mobility by both intuitive analysis and strict mathematical proof.
(3) We analyze the asymptotic expressions on the network capacity. We find that the number of helping nodes can affect the throughput capacity significantly. The network capacity can achieve constant order if the number of helping nodes is large enough, and in this case the time delay can be ensured. We also conclude that the delay constraint *D* affects the throughput capacity. If *D* is not limited, the throughput can also stay constant order, while the time delay can not be ensured.

## 2. Definitions and Notations

The following definitions are presented for the convenience of proof and analysis:

### 2.1. Feasible Aggregate Throughput

If there is a spatial and temporal scheduling strategy that yields an aggregate throughput of *T*(*n*) bits/sec in the networks, we say that the aggregate throughput of *T*(*n*) bits/sec is feasible.

### 2.2. Aggregate Throughput Capacity 

If there exists a constant *c*_1_ < +∞ which satisfies the following inequality, we say that the aggregate capacity is *O*(*f*(*n*)):

$$\underset{n\to +\infty }{\text{lim}}\text{inf}\;P\left(T\left(n\right)=c_{1}f\left(n\right)\;\text{is feasible}\right)<1$$

and if there exist two constants 0 < *c*_2_ < *c*_3_ < +∞ which satisfy the following inequalities, we say that the aggregate throughput capacity is Θ(*f*(*n*)):

$$\underset{n\to +\infty }{\text{lim}}\text{inf}\;P\left(T\left(n\right)=c_{2}f\left(n\right)\;\text{is feasible}\right)=1$$

$$\underset{n\to +\infty }{\text{lim}}\text{inf}\;P\left(T\left(n\right)=c_{3}f\left(n\right)\;\text{is feasible}\right)<1$$

### 2.3. Feasible Per-Node Throughput

If there is a spatial and temporal scheduling strategy that yields a per-node throughput of *λ*(*n*) bits/sec on average, we say that the per-node throughput of *λ*(*n*) bits/sec is feasible.

### 2.4. Per-Node Throughput Capacity

If there exists a constant *c*_4_ < +∞ which satisfies the following inequality, we say that the per-node throughput is *O*(*g*(*n*)):

$$\underset{n\to +\infty }{\text{lim}}\text{inf}\;P\left(\lambda \left(n\right)=c_{4}g\left(n\right)\;\text{is feasible}\right)<1$$

and if there exist two constants 0 < *c*_5_ < *c*_6_ < +∞ which satisfy the following inequalities, we say that the per-node throughput capacity is Θ(*g*(*n*)):

$$\underset{n\to +\infty }{\text{lim}}\text{inf}\;P\left(\lambda \left(n\right)=c_{5}g\left(n\right)\;\text{is feasible}\right)=1$$

$$\underset{n\to +\infty }{\text{lim}}\text{inf}\;P\left(\lambda \left(n\right)=c_{6}g\left(n\right)\;\text{is feasible}\right)<1$$

The following notations are listed throughout this paper. Given two functions *f*(*n*) and *g*(*n*):

(1) *f*(*n*) = *O*(*g*(*n*)) means that there exist positive *c* and *m* so that *f*(*n*) ≤ *cg*(*n*) holds for all *n* ≥ *m*;
(2) *f*(*n*) = Ω(*g*(*n*)) means that there exist positive *c* and *m* so that *f*(*n*) ≥ *cg*(*n*) holds for all *n* ≥ *m*, which also means *g*(*n*) = *O*(*f*(*n*));
(3) *f*(*n*) = Θ(*g*(*n*)) means both *f*(*n*) = *O*(*g*(*n*)) and *f*(*n*) = Ω(*g*(*n*)) hold; *f*(*n*) = *O*(*g*(*n*));
(4) *f*(*n*) = *o*(*g*(*n*)) means lim *f*(*n*)/*g*(*n*) = 0.

The remaining notations are listed in Table 1.

## 3. Network Model

### 3.1. Network Architecture

In our network model, there are *n ad hoc* nodes and *m* helping nodes. The *ad hoc* node spatial distributions are i.i.d., while the helping nodes are regularly placed. Compared with *ad hoc* nodes, the helping nodes are more powerful. They only serve as relay nodes in our network model, and work in routing and transmission of data to other *ad hoc* nodes.

Our network model has a two-tier hierarchy, which is similar to a traditional hybrid network. The difference is that the helping nodes in our network model communicate with each other over wireless channel, whereas the base stations in hybrid networks are connected by a wired network.

**Figure 1:**
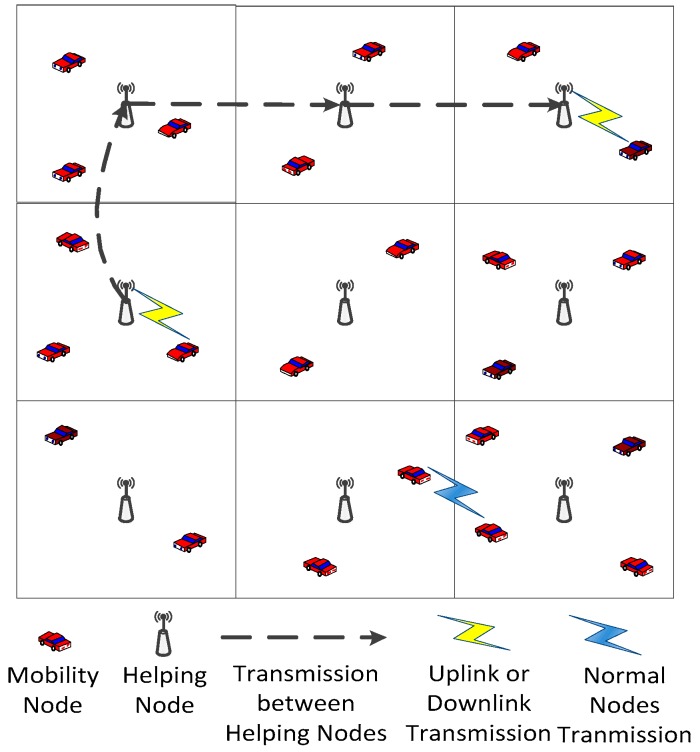
Two-Dimensional Network Architecture.

**Figure 2:**
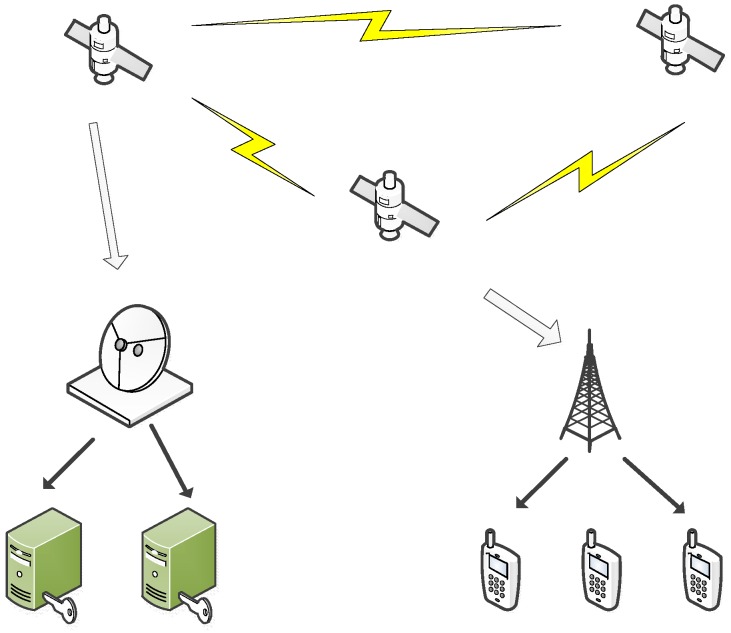
Three-Dimensional Network Architecture.

In this paper we consider two cases: two-dimensional mobile networks and three-dimensional mobile networks. In two-dimensional mobile networks, all the nodes are placed in a two-dimensional square of unit area. In three-dimensional mobile networks, all the nodes are placed in a three-dimensional cube of unit volume. Figure 1 and Figure 2 show the two types of network architecture, respectively.

### 3.2. Communication Model

We adopt the protocol model in [1] as our communication model in this paper. Concretely, let *r* denote the transmission range of *ad hoc* nodes, node *X_j_* can receive the packets from node *X_i_* if the following conditions hold:

(1) Node *X_j_* is under the coverage of node *X_i_*, *i.e.*,
  $$\left|X_{i}-X_{j}\right|\leq r$$
(2) For any other node *X_k_* which is delivering packets over the same time slot,
  $$\left|X_{k}-X_{j}\right|\geq \left(1+\Delta \right)\left|X_{i}-X_{j}\right|$$
  where Δ defines the size of guard zone.

### 3.3. Transmission Strategy

**Figure 3:**
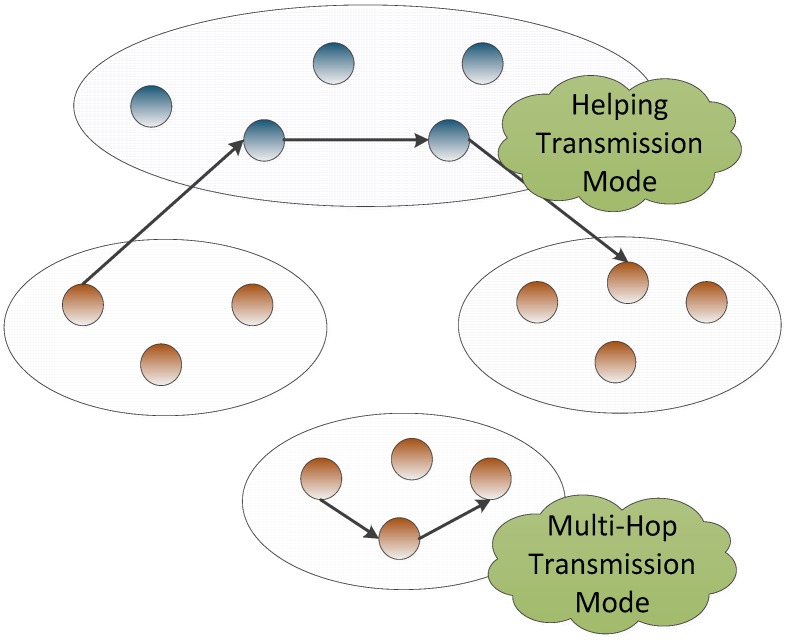
Two Transmission Modes in Networks.

Two transmission modes exist in our network model: helping transmission mode and *ad hoc* transmission mode, as shown in Figure 3. In *ad hoc* transmission mode, packets are delivered from source nodes to destination nodes in a multi-hop fashion, which are relayed by *ad hoc* nodes. In helping transmission mode, packets are first delivered from their source nodes to the backbone network which consists of helping nodes, and then they are transmitted from the helping nodes to their destinations.

In mobile networks, the transmission delay can not be ensured, hence a *D*-delay transmission strategy is considered in this paper. Specifically, a packet will be transmitted by *ad hoc* transmission mode if it can reach its destination node within *D* time slots. Otherwise, the packet will be transmitted by helping transmission mode.

We divide the wireless channel into different sub-channels so that the packet can be carried with different frequency bands. We assume that *ad hoc* nodes have a bandwidth of *W*. We assign *W* into *W*_1_, *W*_2_ and *W*_3_, which denote the intra-cell, uplink and downlink sub-channels, respectively. Hence we have $\sum_{i=1}^{3}W_{i}=W$. Since the amount of traffic carried over uplink sub-channels is the same with that carried over downlink sub-channels, we have *W*_2_ = *W*_3_. Moreover, we assume that the helping nodes have much higher bandwidth than the normal nodes, and the two types of nodes carry packets with different frequency bands, which implies that there is no interference between them.

### 3.4. Mobility Model

Two types of models are considered in this paper.

(1) *Two-dimensional i.i.d. mobility model*: Our two-dimensional mobility model is defined as follows:
  (i) There are *n* mobile *ad hoc* nodes and *m* stable helping nodes distributed in a square of unit are. In each time slot, the *n ad hoc* nodes are uniformly, randomly positioned in the square.
  (ii) The node positions are independent of each other, and independent from time slot to time slot, so the nodes are totally reshuffled in each time slot.
  (iii) There are *n* source-destination pairs in the network. Each node is both a source node and a destination node.
(2) *Three-dimensional i.i.d. mobility model*: Our three-dimensional mobility model is defined as follows:
  (i) There are *n* mobile *ad hoc* nodes and *m* stable helping nodes distributed in a cube of unit volume. In each time slot, the *n ad hoc* nodes are uniformly and randomly positioned in the unit cube.
  (ii) The rest is the same as the two-dimensional i.i.d. mobility model.

In this paper, we consider two time scales of mobility: fast mobility and slow mobility. They are described as follows:

(1) *Fast mobility*: The mobility of nodes is at the same time scale of packet transmission, hence in one time slot, only one-hop transmission is allowed. In this case, the transmission rate (bandwidth) is independent of the number of nodes.
(2) *Slow mobility*: The mobility of nodes is much slower than the packet transmission, hence *W* = Ω(*n*). In this case, the packet size should be W/h(n) to guarantee that *h*(*n*)-hop transmissions are feasible in one time slot.

## 4. Main Results

In this section, we present the main results of this paper. In the study on capacity of mobile networks, many mobility models are proposed to model the mobility of networks. In this paper, we consider four mobility models: two-dimensional i.i.d. fast mobility network model, two-dimensional i.i.d. slow mobility network model, three-dimensional fast mobility network model and three-dimensional slow mobility network model. Our main results are summarized as follows:

(1) Two-dimensional i.i.d. fast mobility network model: it is shown that with a delay constraint *D*, the capacity is $\lambda \left(n,m\right)=O\left(\sqrt{\frac{D}{n}}\right)+\Theta \left(\frac{\sqrt{m}W_{2}}{n}\right)$, where $O\left(\sqrt{\frac{D}{n}}\right)$ is the throughput capacity contributed by *ad hoc* transmission mode and $\Theta \left(\frac{\sqrt{m}W_{2}}{n}\right)$is the capacity contributed by helping transmission mode.
(2) Two-dimensional i.i.d. slow mobility network model: It is shown that with a delay constraint *D*, the capacity is $\lambda \left(n,m\right)=O\left(\sqrt[{3}]{\frac{D}{n}}\right)+\Theta \left(\frac{\sqrt{m}W_{2}}{n}\right)$, where $O\left(\sqrt[{3}]{\frac{D}{n}}\right)$ is the throughput capacity contributed by *ad hoc* transmission mode and $\Theta \left(\frac{\sqrt{m}W_{2}}{n}\right)$ is the capacity contributed by helping transmission mode.
(3) Three-dimensional i.i.d. fast mobility network model: It is shown that with a delay constraint *D*, the capacity is $\lambda \left(n,m\right)=O\left(\sqrt[{3}]{\frac{D}{n^{2}}}\right)+\Theta \left(\frac{\sqrt[{3}]{m^{2}}W_{2}}{n}\right)$, where $O\left(\sqrt[{3}]{\frac{D}{n^{2}}}\right)$ is the capacity contributed by *ad hoc* transmission mode and $\Theta \left(\frac{\sqrt[{3}]{m^{2}}W_{2}}{n}\right)$ is the capacity contributed by helping transmission mode.
(4) Three-dimensional i.i.d. slow mobility network model: It is shown that with a delay constraint *D*, the capacity is $\lambda \left(n,m\right)=O\left(\sqrt[{4}]{\frac{D}{n^{2}}}\right)+\Theta \left(\frac{\sqrt[{3}]{m^{2}}W_{2}}{n}\right)$, where $O\left(\sqrt[{4}]{\frac{D}{n^{2}}}\right)$ is the capacity contributed by *ad hoc* transmission mode and $\Theta \left(\frac{\sqrt[{3}]{m^{2}}W_{2}}{n}\right)$ is the capacity contributed by helping transmission mode.

## 5. Intuitive Analysis

In [38], the authors proposed the virtual channel model. We adopt this idea to have some heuristic arguments on *ad hoc* transmission mode. A virtual channel is a logic representation of a packet transmission from source node to destination node. According to the virtual channel model, there are two main constraints on the network capacity:

(1) Interference: In the process of packet transmissions, the near nodes interfere with each other, which limit the throughput capacity.
(2) Mobility: Since source nodes, destination nodes and relay nodes are mobile all the time, the packets are not ensured to reach their destination nodes before the delay deadline.

**Figure 4:**
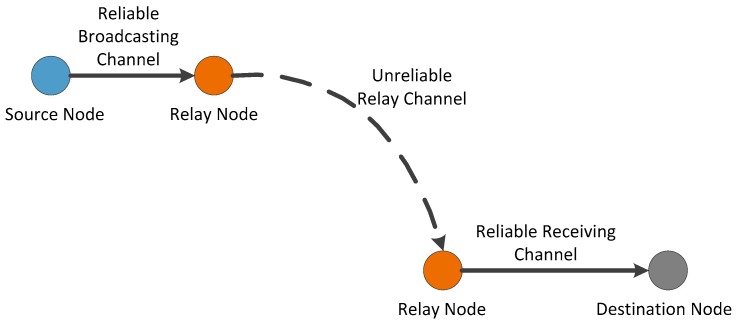
The virtual channel model in *ad hoc* transmission mode.

In a virtual channel mode, a packet is assumed to be transmitted from its source node to its destination node by a two-hop transmission. First, it is delivered from the source node to the relay node around the source. Then, it is delivered from the relay node to the destination node. Hence, in *ad hoc* transmission mode, for a successful packet delivery, we consider three virtual channels, as shown in Figure 4:

*Reliable Broadcasting Channel***:** The source nodes transmit the packets to the relay nodes around it by broadcasting channel;
*Unreliable Relay Channel*: The relay nodes move to the neighborhood of the destination;
*Reliable Receiving Channel*: The relay nodes deliver the packets to the destination nodes.

All the packets delivered to the destinations by *ad hoc* transmission mode are via the three virtual channels. Using the virtual channel model, we present the intuitions on the capacity of our network model.

First the two-dimensional i.i.d. fast mobility model is considered:

*Reliable Broadcasting Channel:* Let *L*_1_ denote the transmission radius of source node. Hence, the source can cover an area of *π*(*L*_1_)^2^. For simplicity, we omit the guard zone Δ. Hence, there are at most $\frac{1}{\pi {\left(L_{1}\right)}^{2}}$ transmitting pairs in the network in a time slot. On average, every source node has *P*_1_ fraction of time to broadcast, where:

$$P_{1}=\frac{1}{\pi {\left(L_{1}\right)}^{2}n}$$

Therefore, for reliable broadcast channel, its throughput is:

$$\frac{W_{1}}{\pi {\left(L_{1}\right)}^{2}n}$$

There exist *π*(*L*_1_)^2^ *n* mobile nodes in a disk with radius *L* on average. Hence, each broadcast can transmit packets *π*(*L*_1_)^2^ *n* nodes as duplicate copies.

*Unreliable Relay Channel:* We assume that the transmission radius for relay nodes is *L*_2_. Since the nodes are distributed in a square of unit area, we assume that *L*_2_ is far less than 1. For a duplicated packet, the probability that it fail to reach the destination node within *D* time slots is:

$$P_{2}={\left(1-\pi {\left(L_{2}\right)}^{2}\right)}^{D}$$

As what analyzed above, a sent packet is delivered to $\pi L_{2}^{2}n$ relay node on average. Hence there are $\pi L_{2}^{2}n$ duplicate copies. Therefore, within *D* time slots, the probability that there are no packets fallen into the area covered by its destination node is:

$$P_{3}={\left(1-\pi {\left(L_{2}\right)}^{2}\right)}^{\pi {\left(L_{1}\right)}^{2}nD}$$

*Reliable Receiving Channel:* Now we consider the packets delivered from relay nodes to destination nodes. Since *L*_2_ is a common transmission radius which all the relay nodes use, the number of transmitting relay-destination pairs in a time slot is no more than $\frac{1}{\pi {\left(L_{2}\right)}^{2}}$. As the highest transmission rate is *W*_1_ for relay nodes, the per-node reliable receiving channel can be expressed by

$$\frac{W_{1}}{\pi {\left(L_{2}\right)}^{2}n}$$

**Figure 5:**
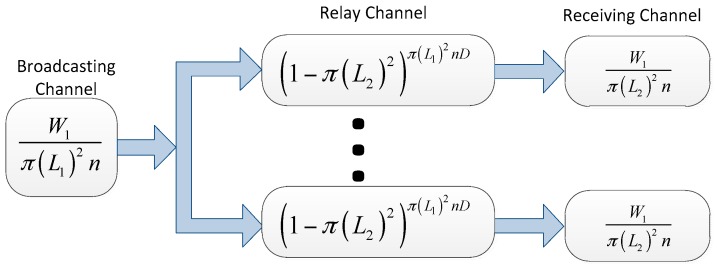
The virtual channel representation for the two-dimensional i.i.d. fast mobility network model.

As shown in Figure 5, for a source-destination pair in the virtual channel model, the maximum throughput is:

$$\lambda =\underset{L_{1},L_{2}}{\text{max}}\;\text{min}\left\{\frac{W_{1}}{\pi {\left(L_{1}\right)}^{2}n}\left(1-{\left(1-\pi {\left(L_{2}\right)}^{2}\right)}^{\pi {\left(L_{1}\right)}^{2}nD}\right),\frac{W_{1}}{\pi {\left(L_{2}\right)}^{2}n}\right\}$$

Next we consider the two-dimensional i.i.d. networks with slow mobility. In virtual channel systems, the relay channel and the receiving channel are the same with the analysis above. Hence we only need to consider the broadcasting channel. For each broadcasting channel, the throughput capacity is:

$$\frac{W_{1}}{\pi L_{1}\sqrt{n}}$$

**Figure 6:**
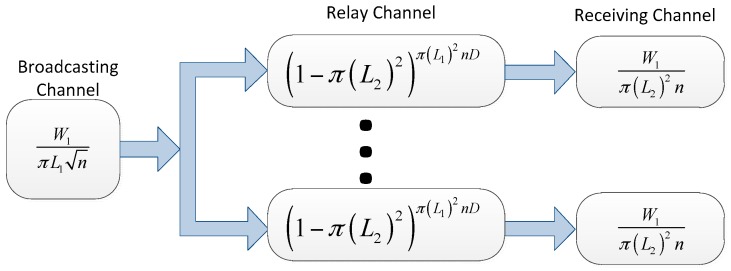
The virtual channel representation for the two-dimensional i.i.d. slow mobility network model.

Hence, the virtual channel systems are as shown in Figure 6:

We can conclude that for a source-destination pair, the maximum throughput is:

$$\lambda =\underset{L_{1},L_{2}}{\text{max}}\;\text{min}\left\{\frac{W_{1}}{\pi L_{1}\sqrt{n}}\left(1-{\left(1-\pi {\left(L_{2}\right)}^{2}\right)}^{\pi {\left(L_{1}\right)}^{2}nD}\right),\frac{W_{1}}{\pi {\left(L_{2}\right)}^{2}n}\right\}$$

Now we consider the three-dimensional i.i.d. fast mobility networks. Different from the analysis above, all the nodes are distributed in a three-dimensional space in this network model. Hence, the broadcasting channel is:

$$\frac{W_{1}}{\frac{4}{3}\pi {\left(L_{1}\right)}^{3}n}$$

The probability that there are no packets fallen into the area covered by its destination node within *D* time slots is:

$${\left(1-\frac{4}{3}\pi {\left(L_{2}\right)}^{3}\right)}^{\frac{4}{3}\pi {\left(L_{1}\right)}^{3}nD}$$

The receiving channel is:

$$\frac{W_{1}}{\frac{4}{3}\pi {\left(L_{2}\right)}^{3}n}$$

**Figure 7:**
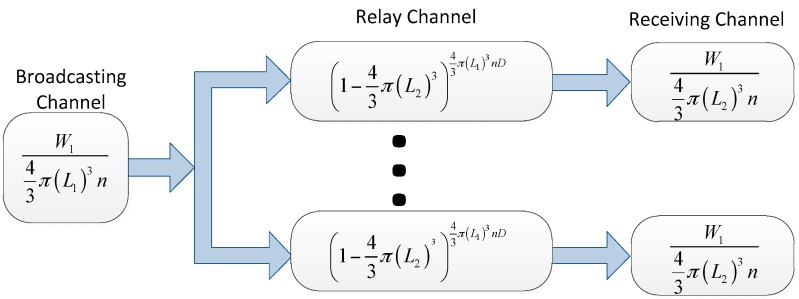
The virtual channel representation for the three-dimensional i.i.d. fast mobility network model.

Figure 7 shows the virtual channel model for three-dimensional i.i.d. fast mobility networks, and the throughput capacity is:

$$\lambda =\underset{L_{1},L_{2}}{\text{max}}\;\text{min}\left\{\frac{W_{1}}{\frac{4}{3}\pi {\left(L_{1}\right)}^{3}n}\left(1-{\left(1-\frac{4}{3}\pi {\left(L_{2}\right)}^{3}\right)}^{\frac{4}{3}\pi {\left(L_{1}\right)}^{3}nD}\right),\frac{W_{1}}{\frac{4}{3}\pi {\left(L_{2}\right)}^{3}n}\right\}$$

Next we turn to the three-dimensional i.i.d. slow mobility network model. The relay channel and the receiving channel have been obtained in the analysis of three-dimensional fast mobility networks. As described in [38], the broadcasting channel in three-dimensional slow mobility can be expressed by:

$$\frac{W_{1}}{\frac{4}{3}\pi L_{1}\sqrt[{3}]{n}}$$

**Figure 8:**
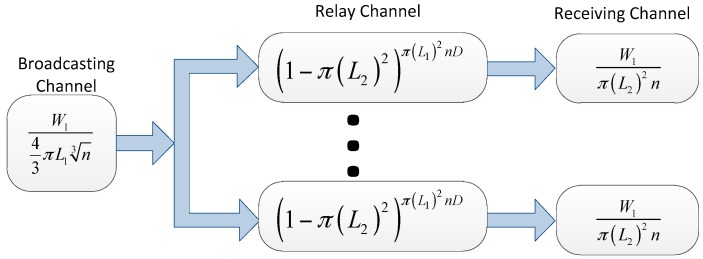
The virtual channel representation for the three-dimensional i.i.d. slow mobility network model.

As shown in Figure 8, we can obtain the throughput capacity:

$$\lambda =\underset{L_{1},L_{2}}{\text{max}}\;\text{min}\left\{\frac{W_{1}}{\frac{4}{3}\pi L_{1}\sqrt[{3}]{n}}\left(1-{\left(1-\frac{4}{3}\pi {\left(L_{2}\right)}^{3}\right)}^{\frac{4}{3}\pi {\left(L_{1}\right)}^{3}nD}\right),\frac{W_{1}}{\frac{4}{3}\pi {\left(L_{2}\right)}^{3}n}\right\}$$

## 6. Capacity of Heterogeneous Mobile Wireless Networks

In this section we calculate the capacity of the four types of mobile network model, respectively. First we derive the capacity contributed by *ad hoc* transmission mode. Then we consider the capacity contributed by helping transmission mode. To obtain the throughput contributed by *ad hoc* transmission mode, we first assume that relaying is not allowed in the networks and calculate the number of packets delivered directly from the source to the destination. On this basis, we derive the number of packets delivered with the help of relay nodes. Summing up the two results, we can get the total number of packets carried by *ad hoc* transmission mode. To obtain the throughput capacity contributed by helping transmission mode, we should consider the network capacity of the backbone network, which has regular topology architecture.

### 6.1. Capacity of Two-Dimensional i.i.d. Fast Mobility Networks

We will first present some important inequalities firstly, which are shown in the following theorem:

**Theorem** **1.**  *In two-dimensional mobile networks, we have the following inequalities:*

(1) $$\Lambda [T]\leq nW_{1}T$$

(2) $$\left|R[T]\right|\leq nW_{1}T$$

(3) $$\sum_{B=1}^{\Lambda [T]}\frac{\Delta^{2}}{16}{\left(\alpha_{B}\right)}^{2}\leq \frac{W_{1}T}{\pi }$$

**Proof.**  For one node, the largest number of bits it can deliver in one time slot is *W*_1_ bits. Therefore, in *T* time slots, all the nodes in the network can take at most *nW*_1_*T* bits, which are necessarily larger than the packets successfully transmitted to the destination nodes from source nodes, as shown in inequality Equation (1). It is also larger than the number of bits which are stored at relay nodes. Hence we have equality Equation (2). □

Now we investigate the throughput capacity contributed by *ad hoc* transmission mode. First we will analyze the number of bits directly transmitted to the source nodes without relaying in *T* time slots, which is denoted by Λ^*d*^[*T*]. We have the following theorem:

**Theorem** **2.**  *In two-dimensional i.i.d fast mobility model, without considering relaying progress, we can bound the number of bits directly delivered to destination nodes as:*

$$E\left[\Lambda^{d}[T]\right]\leq \frac{8\sqrt{2}}{\Delta }W_{1}T\sqrt{n}$$

**Proof.**  Consider inequality Equation (3), according to the Cauchy-Schwarz inequality, we have:

$$\begin{array}{ll} {\left(\sum_{B=1}^{\Lambda^{d}[T]}\left(\alpha_{B}\right)\right)}^{2} & \leq \left(\sum_{B=1}^{\Lambda^{d}[T]}1\right)\left(\sum_{B=1}^{\Lambda^{d}[T]}{\left(\alpha_{B}\right)}^{2}\right)\\ & \leq \Lambda^{d}[T]\frac{16WT}{\pi \Delta^{2}} \end{array}$$

which can be expressed by:

(4) $$E\left[\sum_{B=1}^{\Lambda^{d}[T]}\alpha_{B}\right]\leq E\left[\sqrt{\Lambda^{d}[T]}\right]\left(\sqrt{\frac{16WT}{\Delta^{2}\pi }}\right)$$

The inequality above gives a bound on the expected distance which the bits take. For two arbitrary nodes *X_i_* and *X_j_*, let *dist*(*X_i_*, *X_j_*)(*t*) denote their distance in time slot *t*. Based on the assumption that the mobility model are i.i.d, we have:

$$\text{Pr}\left(dist(X_{i},X_{j})(t)\leq L\right)=\pi L^{2}$$

In *T* time slots, we have:

$$E\left[\sum_{t=1}^{T}\left(\sum_{i=1}^{n-1}1_{dist(X_{1},X_{i+1})(t)\leq L}\right)\right]=Tn\pi L^{2}$$

The network can carry at most *W*_1_ bits in single time slot, hence we can obtain:

$$\sum_{B=1}^{\Lambda^{d}[T]}1_{\alpha_{B}\leq L}\leq W_{1}\sum_{t=1}^{T}\sum_{i=1}^{n-1}1_{dist(X_{i},X_{i+1})(t)\leq L}$$

According to the inequalities above, we have:

(5) $$E\left[\Lambda^{d}[T]\right]-E\left[\sum_{B=1}^{\Lambda^{d}[T]}1_{\alpha_{B}>L}\right]\leq W_{1}\pi nTL^{2}$$

By Jensen’s inequality, combining inequality Equation (4) and inequality Equation (5), we can obtain the following inequality:

$$\begin{array}{ll} \sqrt{E\left[\Lambda^{d}[T]\right]\frac{16WT}{\Delta^{2}\pi }} & \geq E\left[\sqrt{\Lambda^{d}[T]}\right]\left(\sqrt{\frac{16WT}{\Delta^{2}\pi }}\right)\\ & \geq E\left[\sum_{B=1}^{\Lambda^{d}[T]}\alpha_{B}\right]\\ & \geq E\left[\sum_{B=1}^{\Lambda^{d}[T]}1_{\alpha_{B}>L}\right]L\\ & \geq \left(E\left[\Lambda^{d}[T]\right]-W_{1}\pi nTL^{2}\right)L \end{array}$$

where Jensen’s inequality is used in the first inequality.

Let $L=\sqrt{\frac{E\left[\Lambda^{d}[T]\right]}{2\pi W_{1}nT}}$, we obtain:

$$\sqrt{\frac{16W_{1}T}{\Delta^{2}\pi }E\left[\Lambda^{d}[T]\right]}\geq \frac{1}{2}E\left[\Lambda^{d}[T]\right]\sqrt{\frac{E\left[\Lambda^{d}[T]\right]}{2\pi W_{1}nT}}$$

Simplify the inequality above, we can obtain *E*[Λ^*d*^[*T*]]. □

Theorem 2 has given number of bits directly delivered to destination nodes. Now we consider the scenario where packets can be relayed in the networks.

**Theorem** **3.**  *In two-dimensional i.i.d fast mobility model, if packet relaying exists, the bound on the number of bits transmitted to the destination number through relay nodes is:*

$$E\left[\Lambda^{r}[T]\right]\leq \frac{8\sqrt{2}}{\Delta }W_{1}T\sqrt{nD}$$

where *D* is delay constant.

**Proof.**  In *T* time slots, let Λ^*r*^[*T*] denote the number of bits which are transmitted from relay nodes to destination nodes in *T* time slots, we have Λ[*T*] = Λ^*r*^[*T*] + Λ^*d*^[*T*]. According to Cauchy-Schwarz inequality and inequality Equation (3), we obtain:

$$\begin{array}{ll} {\left(\sum_{B=1}^{\Lambda^{r}[T]}\left(\alpha_{B}\right)\right)}^{2} & \leq \left(\sum_{B=1}^{\Lambda^{r}[T]}1\right)\left(\sum_{B=1}^{\Lambda^{r}[T]}{\left(\alpha_{B}\right)}^{2}\right)\\ & \leq \Lambda^{r}[T]\frac{16WT}{\pi \Delta^{2}} \end{array}$$

where Cauchy-Schwarz inequality is used in the first inequality. Hence we have:

(6) $$E\left[\sum_{B=1}^{\Lambda^{r}[T]}\alpha_{B}\right]\leq E\left[\sqrt{\Lambda^{r}[T]}\right]\left(\sqrt{\frac{16WT}{\Delta^{2}\pi }}\right)$$

Consider bit *B*, let *d_B_* denote its destination node and *e_B_* denote the node storing bit *B*. We assume that from time slot *t_B_* to time slot *t_B_* + *D* − 1, the minimum distance between *d_B_* and *e_B_* is *L_B_*. Then we have:

$$L_{B}=\underset{t_{B}\leq t\leq D+t_{B}-1}{\text{min}}dist(d_{B},e_{B})(t)$$

For an arbitrary bit *B* which is in the set *R*[*T*], we have:

$$\text{Pr}\left(L_{B}\leq L\right)=1-{\left(1-\pi L^{2}\right)}^{D}$$

According to Taylor’s formula, equality Equation (14) can be expressed as:

$$\text{Pr}\left(L_{B}\leq L\right)=\pi DL^{2}+o(\pi DL^{2})\leq \pi DL^{2}$$

Therefore, we obtain:

(7) $$E\left[\sum_{B\in R[T]}1_{L_{B}\leq L}\right]\leq nW_{1}T\pi DL^{2}$$

Obviously, we have:

(8) $$\sum_{B\in R[T]}1_{L_{B}\leq L}\geq \sum_{B=1}^{\Lambda^{r}[T]}1_{\alpha_{B}\leq L}$$

Combining Equations (7) and (8), we have:

$$E\left[\sum_{B=1}^{\Lambda^{r}[T]}1_{\alpha_{B}\leq L}\right]\leq nW_{1}T\pi DL^{2}$$

Hence, we can obtain the following inequalities:

(9) $$\begin{array}{ll} E\left[\sum_{B=1}^{\Lambda^{r}[T]}\alpha_{B}\right] & \geq E\left[\sum_{B=1}^{\Lambda^{r}[T]}\alpha_{B}1_{\alpha_{B}>L}\right]\\ & \geq E\left[\sum_{B=1}^{\Lambda^{r}[T]}1_{\alpha_{B}>L}\right]L\\ & \geq \left(E\left[\Lambda^{r}[T]\right]-E\left[\sum_{B=1}^{\Lambda^{r}[T]}1_{\alpha_{B}\leq L}\right]\right)L\\ & \geq E\left[\Lambda^{r}[T]\right]L-nW_{1}T\pi DL^{3} \end{array}$$

According to Jensen's inequality, combining inequality Equation (6) and inequality Equation (9), we have:

$$\begin{array}{ll} E\left[\sqrt{\Lambda^{r}[T]}\right]\left(\sqrt{\frac{16W_{1}T}{\Delta^{2}\pi }}\right) & \geq E\left[\sqrt{\frac{16W_{1}T}{\Delta^{2}\pi }\Lambda^{r}[T]}\right]\\ & \geq E\left[\sum_{B=1}^{\Lambda^{r}[T]}\alpha_{B}\right]\\ & \geq E\left[\Lambda^{r}[T]\right]L-nW_{1}T\pi DL^{3} \end{array}$$

where Jensen’s inequality is used in the first inequality.

Let $L=\sqrt{\frac{E\left[\Lambda^{r}[T]\right]}{2\pi W_{1}nTD}}$, we can have *E*[Λ^*r*^[*T*]]. □

**Theorem** **4.**  *In two-dimensional i.i.d. fast mobility networks, the ad hoc transmission mode capacity is:*

$$\lambda_{A}\left(n\right)=O\left(\sqrt{\frac{D}{n}}\right)$$

**Proof.**  Combining Theorems 2 and 3, we have:

$$E\left[\Lambda [T]\right]\leq \frac{8\sqrt{2}}{\Delta }W_{1}T\sqrt{n}\left(1+\sqrt{D}\right)$$

In the meaning of order, the throughput capacity contributed by relaying nodes plays a dominating role compared with the throughput without relaying node. Thus, we have $E\left[\frac{\Lambda \left[T\right]}{n}\right]\leq O\left(\frac{\sqrt{nD}}{n}\right)$. □

Now we consider the capacity contributed by the helping nodes. In two-dimensional network model, the helping nodes are regularly placed and divide the area into (1m)×(1m) equal-size squares. In the transmissions of helping nodes, there are three phases: uplink phase, downlink phase and HN-to-HN phase. In uplink phase, the source nodes deliver the packets to the nearest helping nodes. In this case we call the helping nodes 'sources'. Then, the helping nodes transmit the packets to the helping nodes that are in the same cells with the destination nodes. In this case we call the helping nodes 'destinations'. Finally, the helping nodes deliver the packets to the destination nodes by broadcasting. Theorem 5 gives the capacity contributed by the backbone network which consists of helping nodes.

**Theorem** **5.**  *In two-dimensional networks, the per-node throughput helping transmission mode contributes is:*

$$\lambda_{H}\left(m,n\right)=\Theta \left(\frac{\sqrt{m}W_{2}}{n}\right)$$

**Proof.**  Since the node distribution is i.i.d., each helping node is chosen as a source or a destination with the same probability. We randomly choose the helping node on (*p*, *q*) as the source and the helping node on (*i*, *j*) as the destination. In our network model, two neighbor helping nodes can communicate directly, hence the minimum number of hops is *h* = |*p* − *i*| + |*j* − *q*|. Summing up all the possible *h*, we have:

$$\begin{array}{ll} E\left[h\right] & =E\left[\left|p-i\right|\right]+E\left[\left|p-i\right|\right]\\ & =2E\left[E\left[\left|p-i\right|\right]\right] \end{array}$$

*E*[|*p* − *i*|] can be obtained by the following equation:

(10) $$\begin{array}{ll} E\left[\left|p-i\right|\right] & =\frac{1}{\sqrt{m}}\left(\sum_{i=1}^{p-1}\left(p-i\right)+\sum_{i=p}^{\sqrt{m}}\left(i-p\right)\right)\\ & =\frac{p^{2}}{\sqrt{m}}-\left(\frac{1}{\sqrt{m}}+1\right)p+\frac{\sqrt{m}+1}{2} \end{array}$$

We can get *E*[*p*^2^] by the following equation:

(11) $$\begin{array}{ll} E\left[p^{2}\right] & =\frac{1^{2}+2^{2}+\ldots +{\left(\sqrt{m}\right)}^{2}}{\sqrt{m}}\\ & =\frac{\left(\sqrt{m}+1\right)\left(2\sqrt{m}+1\right)}{6} \end{array}$$

Since *p* is uniformly distributed in the interval $\left[1,\sqrt{m}\right]$, we obtain:

(12) $$E\left[p\right]=\frac{1+\sqrt{m}}{2}$$

Substituting Equations (11) and (12) into Equation (10), we have:

(13) $$E\left[h\right]=\sqrt{m}-\frac{1}{\sqrt{m}}$$

Since the bandwidth of uplink sub-channel is *W*_2_, for each helping node, it can carry at most *W*_2_ bits in each time slot. Thus, we have:

(14) $$n\lambda_{H}\bar{h}m\leq mW_{2}$$

where *λ_H_* is the per-node throughput capacity of backbone helping node.

For an arbitrary helping node *Y_i_*, its interference region is a circle with radius (1 + Δ_*H*_)*r_H_*. Each helping node occupies a region of ${\left(\frac{1}{\sqrt{m}}\right)}^{2}$ in the unit area, hence the number of helping nodes interfered by *Y_i_* is:

$$c_{7}=\frac{\pi {\left(\left(1+\Delta_{H}\right)r_{H}\right)}^{2}}{{\left(\frac{1}{\sqrt{m}}\right)}^{2}}\approx \pi {\left(1+\Delta_{H}\right)}^{2}$$

Hence the number of nodes interfered by *Y_i_* is bounded by *c*_8_ = *c*_7_ − 1. It implies that there exists a temporal and spatial scheduling strategy that every helping node can obtain at least one slot to communicate in every (1 + *c*_8_) slots. Therefore, we have:

(15) $$n\lambda_{H}\bar{h}m\geq \frac{mW_{2}T}{2\left(1+c_{8}\right)}$$

Combining Equations (14) and (15), we can obtain the per-node throughput capacity of backbone:

$$\lambda_{H}\left(m\right)=\Theta \left(\frac{\sqrt{m}W_{2}}{n}\right)$$

□

Since the total throughput capacity is contributed by multi-hop mode and helping mode, we have *λ*(*n*, *m*) = *λ_A_*(*n*) + *λ_H_*(*m*). According to Theorems 4 and 5, we can obtain Theorem 6 as follows:

**Theorem** **6.**  *In two-dimensional i.i.d. fast mobility networks, under protocol model, the network capacity is:*

$$\lambda \left(n,m\right)=O\left(\sqrt{\frac{D}{n}}\right)+\Theta \left(\frac{\sqrt{m}W_{2}}{n}\right)$$

**Proof.**  Combining Theorems 4 and 5, we can prove the theorem above. □

### 6.2. Capacity of Two-Dimensional i.i.d. Slow Mobility Networks

Similar to the fast mobility model, we can calculate the throughput capacity of slow mobility model. First we present some important inequalities, as shown in Theorem 7:

**Theorem** **7.**  *In two-dimensional i.i.d. slow mobility networks, we have the following inequalities:*

(16) $$\sum_{B=1}^{\Lambda [T]}\sum_{h=1}^{H(B)}1\leq nW_{1}T$$

(17) $$\sum_{B=1}^{\Lambda [T]}\sum_{h=1}^{H(B)}\frac{\Delta^{2}}{16}{\left(\alpha_{B}^{h}\right)}^{2}\leq \frac{W_{1}T}{\pi }$$

**Proof.**  Similarly with the proof of Theorem 1, we can prove the theorem above. □

First we consider the case that all the packets are directly transmitted to the destination nodes without relaying.

**Theorem** **8.**  *In two-dimensional i.i.d. slow mobility networks, if relaying progress is not allowed, the number of bits directly delivered to destination nodes is bounded as follows:*

$$E\left[\Lambda^{d}[T]\right]\leq \frac{4\sqrt[{3}]{2}}{\sqrt[{3}]{\Delta^{2}}}W_{1}T\sqrt[{3}]{n^{2}}$$

**Proof.**  According to Theorem 1 and the Cauchy-Schwarz inequality, we have:

$$\begin{array}{ll} {\left(\sum_{B=1}^{\Lambda [T]}\sum_{h=1}^{H(B)}\alpha_{B}^{h}\right)}^{2} & \leq \left(\sum_{B=1}^{\Lambda [T]}\sum_{h=1}^{H(B)}1\right)\left(\sum_{B=1}^{\Lambda [T]}\sum_{h=1}^{H(B)}{\left(\alpha_{B}^{h}\right)}^{2}\right)\\ & \leq nW_{1}T\sum_{B=1}^{\Lambda [T]}\sum_{h=1}^{H(B)}{\left(\alpha_{B}^{h}\right)}^{2}\\ & \leq \frac{16nW_{1}^{2}T^{2}}{\Delta^{2}\pi } \end{array}$$

where the Cauchy-Schwarz inequality is used in the first inequality.

Since $\sum_{h=1}^{H(B)}\alpha_{B}^{h}\geq L_{B}$, we have that:

(18) $$\sum_{B=1}^{\Lambda [T]}L_{B}\leq \frac{4\sqrt{n}W_{1}T}{\Delta \sqrt{\pi }}$$

Similar to the analysis in Theorem 2, the following inequality can be obtained:

(19) $$E\left[\Lambda^{d}[T]\right]-E\left[\sum_{B=1}^{\Lambda^{d}[T]}1_{L_{B}>L}\right]\leq W_{1}\pi nTL^{2}$$

Combining inequality Equation (18) and inequality Equation (19), we have:

$$\begin{array}{ll} \frac{4\sqrt{n}W_{1}T}{\Delta \sqrt{\pi }} & \geq E\left[\sum_{B=1}^{\Lambda^{d}[T]}L_{B}\right]\\ & \geq LE\left[\sum_{B=1}^{\Lambda^{d}[T]}1_{L_{B}>L}\right]\\ & \geq L\left(E\left[\Lambda^{d}[T]\right]-W_{1}\pi nTL^{2}\right) \end{array}$$

Let $L=\sqrt{\frac{E\left[\Lambda^{d}[T]\right]}{2\pi W_{1}nT}}$, we can conclude that:

$$\frac{1}{2}E\left[\Lambda^{d}[T]\right]\sqrt{\frac{E\left[\Lambda^{d}[T]\right]}{2\pi W_{1}nT}}\leq \frac{4\sqrt{n}W_{1}T}{\Delta \sqrt{\pi }}$$

Simplify the inequality above, we can obtain Theorem 8. □

**Theorem** **9.**  *In two-dimensional i.i.d. slow mobility network model, if packet relaying is allowed, the bound on the number of bits which are transmitted successfully from relay nodes to destination nodes is as follows:*

$$E\left[\Lambda^{r}[T]\right]\leq \frac{4\sqrt[{3}]{2}}{\sqrt[{3}]{\Delta^{2}}}W_{1}T\sqrt[{3}]{n^{2}D}$$

**Proof.**  Similar to the steps in capacity analysis of network capacity of two-dimensional i.i.d. fast mobility networks, we have:

$$E\left[\sum_{B=1}^{\Lambda^{r}[T]}1_{L_{B}\leq L}\right]\leq nW_{1}T\pi DL^{2}$$

Therefore, we can obtain the following inequalities:

(20) $$\begin{array}{ll} E\left[\sum_{B=1}^{\Lambda^{r}[T]}L_{B}\right] & \geq E\left[\sum_{B=1}^{\Lambda^{r}[T]}L_{B}1_{L_{B}>L}\right]\\ & \geq E\left[\sum_{B=1}^{\Lambda^{r}[T]}1_{L_{B}>L}\right]L\\ & \geq \left(E\left[\Lambda^{r}[T]\right]-E\left[\sum_{B=1}^{\Lambda^{r}[T]}1_{L_{B}<L}\right]\right)L\\ & \geq E\left[\Lambda^{r}[T]\right]L-nW_{1}T\pi DL^{3} \end{array}$$

Combining inequality Equation (18) and inequality Equation (20), we have:

$$\frac{4\sqrt{n}W_{1}T}{\Delta \sqrt{\pi }}\geq E\left[\Lambda^{r}[T]\right]L-nW_{1}T\pi DL^{3}$$

Let $L=\sqrt{\frac{E\left[\Lambda^{r}[T]\right]}{2\pi W_{1}nTD}}$, we have Theorem 9. □

**Theorem** **10.**  *In two-dimensional i.i.d. slow mobility networks, the throughput capacity contributed by ad hoc transmission mode is:*

(21) $$\lambda_{A}\left(n\right)=O\left(\sqrt[{3}]{\frac{D}{n}}\right)$$

**Proof.**  Combining Theorems 9 and 10, we have:

$$E\left[\Lambda [T]\right]\leq \frac{4\sqrt[{3}]{2}}{\sqrt[{3}]{\Delta^{2}}}W_{1}T\sqrt[{3}]{n^{2}}\left(1+\sqrt[{3}]{D}\right)$$

Inequality Equation (21) then follows the proof of Theorem 4. □

Theorem 5 has given the throughput capacity contributed by backbone networks. Combining the results of Theorems 5 and 10, we can obtain the network capacity of two-dimensional i.i.d. slow mobility networks:

**Theorem** **11.**  *In two-dimensional i.i.d. slow mobility networks, under protocol model, the capacity is:*

$$\lambda \left(n,m\right)=O\left(\sqrt[{3}]{\frac{D}{n}}\right)+\Theta \left(\frac{\sqrt{m}W_{2}}{n}\right)$$

**Proof.**  Combining Theorems 5 and 10, we can prove the theorem above. □

### 6.3. Capacity of Three-Dimensional i.i.d. Fast Mobility Networks

Inequality Equation (1) and inequality Equation (2) which hold in two-dimensional networks are also suitable for three-dimensional networks. However, in three-dimensional i.i.d fast mobility model, we should modify inequality Equation (3) to analyze the network capacity of the networks.

**Theorem** **12.**  *In three-dimensional i.i.d fast mobility model, we have:*

(22) $$\sum_{B=1}^{\Lambda [T]}\frac{\Delta^{3}}{24}{\left(\alpha_{B}\right)}^{3}\leq \frac{W_{1}T}{\pi }$$

**Proof.**  The proof progress is similar with Theorem 1. □

**Theorem** **13.**  *In three-dimensional i.i.d fast mobility model, we can obtain that the bounds on the number of bits directly delivered to destination nodes by ad hoc transmission mode:*

$$E\left[\Lambda^{d}[T]\right]\leq \frac{6\sqrt[{3}]{6}}{\Delta }\sqrt[{3}]{W_{1}^{2}T^{2}n}$$

**Proof.**  According to the Cauchy-Schwarz inequality, we can obtain the following inequality:

$$\begin{array}{ll} {\left(\sum_{B=1}^{\Lambda^{d}[T]}\alpha_{B}\right)}^{3} & \leq \left(\sum_{B=1}^{\Lambda^{d}[T]}1\right)\left(\sum_{B=1}^{\Lambda^{d}[T]}\alpha_{B}^{3}\right)\\ & \leq \Lambda^{d}[T]\frac{24W_{1}T}{\pi \Delta^{3}} \end{array}$$

which implies:

(23) $$E\left[\sum_{B=1}^{\Lambda^{d}[T]}\alpha_{B}\right]\leq \sqrt[{3}]{\Lambda^{d}[T]}\left(\sqrt[{3}]{\frac{24W_{1}T}{\pi \Delta^{3}}}\right)$$

Since the node distribution is i.i.d in a three-dimensional space, according to the knowledge of Probability Theory, we have that:

$$\text{Pr}\left(dist(X_{i},X_{j})(t)\leq L\right)=\frac{4\pi }{3}L^{3}$$

According to the inequality above, we conclude that:

$$E\left[\sum_{t=1}^{T}\left(\sum_{i=1}^{n-1}1_{dist(X_{1},X_{i+1})(t)\leq L}\right)\right]=\frac{4\pi }{3}TnL^{3}$$

In single time slot, each node can carry at most *W*_1_ bits. Hence we have:

$$\sum_{B=1}^{\Lambda^{d}[T]}1_{\alpha_{B}\leq L}\leq W_{1}\sum_{t=1}^{T}\sum_{i=1}^{n-1}1_{dist(X_{i},X_{i+1})(t)\leq L}$$

Taking expectation on the inequality above, we have:

(24) $$E\left[\Lambda^{d}[T]\right]-E\left[\sum_{B=1}^{\Lambda^{d}[T]}1_{\alpha_{B}>L}\right]\leq \frac{4\pi }{3}W_{1}nTL^{3}$$

Combining inequality Equation (23) and inequality Equation (24), we have:

$$\begin{array}{ll} \sqrt[{3}]{E\left[\Lambda^{d}[T]\right]\frac{24WT}{\Delta^{3}\pi }} & \geq E\left[\sqrt[{3}]{\Lambda^{d}[T]}\right]\left(\sqrt[{3}]{\frac{24WT}{\Delta^{3}\pi }}\right)\\ & \geq E\left[\sum_{B=1}^{\Lambda^{d}[T]}\alpha_{B}\right]\\ & \geq E\left[\sum_{B=1}^{\Lambda^{d}[T]}1_{\alpha_{B}>L}\right]L\\ & \geq \left(E\left[\Lambda^{d}[T]\right]-\frac{4\pi }{3}W_{1}nTL^{3}\right)L \end{array}$$

where Jensen’s inequality is used in the first inequality.

Let $L=\sqrt[{3}]{\frac{E\left[\Lambda^{d}[T]\right]}{2\pi W_{1}nT}}$, we have:

$$\sqrt[{3}]{\frac{24W_{1}T}{\Delta^{3}\pi }E\left[\Lambda^{d}[T]\right]}\geq \frac{1}{3}E\left[\Lambda^{d}[T]\right]\sqrt[{3}]{\frac{E\left[\Lambda^{d}[T]\right]}{2\pi W_{1}nT}}$$

Then Theorem 14 can be proved by simplifying the inequality above. □

Next we study the throughput contributed by *ad hoc* mode considering relaying progress.

**Theorem** **14.**  *In three-dimensional i.i.d fast mobility networks, if relaying exists, we can obtain the bound on the number of bits successfully delivered to the destination nodes through relay nodes as follows:*

$$E\left[\Lambda^{r}[T]\right]\leq \frac{6\sqrt[{3}]{6}}{\Delta }\sqrt[{3}]{W_{1}^{2}T^{2}Dn}$$

where *D* is the delay constant.

**Proof.**  According to the Cauchy-Schwarz inequality and inequality Equation (48), we have the following inequalities:

$$\begin{array}{ll} {\left(\sum_{B=1}^{\Lambda^{r}[T]}\alpha_{B}\right)}^{3} & \leq \left(\sum_{B=1}^{\Lambda^{r}[T]}1\right)\left(\sum_{B=1}^{\Lambda^{r}[T]}\alpha_{B}^{3}\right)\\ & \leq \Lambda^{r}[T]\frac{24W_{1}T}{\pi \Delta^{3}} \end{array}$$

which can be expressed by:

(25) $$E\left[\sum_{B=1}^{\Lambda^{r}[T]}\alpha_{B}\right]\leq \sqrt[{3}]{\Lambda^{r}[T]}\left(\sqrt[{3}]{\frac{24W_{1}T}{\pi \Delta^{3}}}\right)$$

Consider bit *B*, in time [*t_B_*, *D* + *t_B_* − 1], let $L_{B}^{\prime }$ denote the minimum distance between its relay node *e_B_* and destination node *d_B_*, *i.e.*:

$$L_{B}^{\prime }=\underset{t_{B}\leq t\leq D+t_{B}-1}{\text{min}}dist(d_{B},e_{B})(t)$$

Since the node distribution is i.i.d in each time slot, we have:

$$\text{Pr}\left(L_{B}^{\prime }\leq L\right)=1-{\left(1-\frac{4\pi }{3}L^{3}\right)}^{D}$$

Using the Taylor formula, we can obtain the following inequality:

$$\text{Pr}\left(L_{B}^{\prime }\leq L\right)=\frac{4\pi }{3}DL^{3}+o(\frac{4\pi }{3}DL^{3})\leq \frac{4\pi }{3}DL^{3}$$

Then we can get the bound on the expectation of the number of bits which satisfy $L_{B}^{\prime }\leq L$:

$$E\left[\sum_{B\in R[T]}1_{L_{B}^{\prime }\leq L}\right]\leq \frac{4\pi }{3}nW_{1}TDL^{3}$$

Since $\sum_{B\in R[T]}1_{L_{B}^{\prime }\leq L}\geq \sum_{B=1}^{\Lambda^{r}[T]}1_{\alpha_{B}\leq L}$, we have:

$$E\left[\sum_{B=1}^{\Lambda^{r}[T]}1_{\alpha_{B}\leq L}\right]\leq \frac{4\pi }{3}nW_{1}TDL^{3}$$

Furthermore, we have:

(26) $$\begin{array}{ll} E\left[\sum_{B=1}^{\Lambda^{r}[T]}\alpha_{B}\right] & \geq E\left[\sum_{B=1}^{\Lambda^{r}[T]}\alpha_{B}1_{\alpha_{B}>L}\right]\\ & \geq E\left[\sum_{B=1}^{\Lambda^{r}[T]}1_{\alpha_{B}>L}\right]L\\ & \geq \left(E\left[\Lambda^{r}[T]\right]-E\left[\sum_{B=1}^{\Lambda^{r}[T]}1_{\alpha_{B}\leq L}\right]\right)L\\ & \geq E\left[\Lambda^{r}[T]\right]L-\frac{4\pi }{3}nW_{1}TDL^{4} \end{array}$$

Combining inequality Equation (25) and inequality Equation (26), we can obtain the following inequalities:

$$\begin{array}{ll} E\left[\sqrt{\Lambda^{r}[T]}\right]\left(\sqrt{\frac{24W_{1}T}{\Delta^{3}\pi }}\right) & \geq E\left[\sqrt{\frac{24W_{1}T}{\Delta^{3}\pi }\Lambda^{r}[T]}\right]\\ & \geq E\left[\sum_{B=1}^{\Lambda^{r}[T]}\alpha_{B}\right]\\ & \geq E\left[\Lambda^{r}[T]\right]L-\frac{4\pi }{3}nW_{1}TDL^{4} \end{array}$$

where Jensen’s inequality is used in the first inequality.

Let $L=\sqrt[{3}]{\frac{E\left[\Lambda^{r}[T]\right]}{2\pi W_{1}nT}}$, we can prove Theorem 14. □

Combining Theorems 13 and 14, we can obtain the capacity contributed by *ad hoc* transmission mode:

**Theorem** **15.**  *In three-dimensional i.i.d. fast mobility networks, the ad hoc transmission mode capacity is:*

$$\lambda_{A}\left(n\right)=O\left(\sqrt[{3}]{\frac{D}{n^{2}}}\right)$$

**Proof.**  Based on Theorems 13 and 14, we have the total number of bits transmitted by *ad hoc* transmission mode as follows:

$$E\left[\Lambda [T]\right]\leq \frac{6\sqrt[{3}]{6}}{\Delta }\sqrt[{3}]{W_{1}^{2}T^{2}n}\left(\sqrt[{3}]{D}+1\right)$$

According to the definition of order, we can obtain the capacity contributed by *ad hoc* transmission mode:

$$E\left[\frac{\Lambda \left[T\right]}{n}\right]\leq O\left(\frac{\sqrt[{3}]{nD}}{n}\right)$$

Now we study the throughput capacity contributed by the helping nodes, which can be obtained by the following theorem:

**Theorem** **16.**  *In three-dimensional networks, the per-node throughput capacity contributed by the backbone network is:*

$$\lambda_{H}\left(m\right)=\Theta \left(\frac{\sqrt[{3}]{m^{2}}W_{2}}{n}\right)$$

**Proof.**  In three-dimensional backbone network, we randomly choose the helping node on (*p*, *q*, *u*) and the helping node on (*i*, *j*, *v*) as source and destination respectively. Hence we have that the expectation of the hops is *E*[*h*] = 3*E*[*E*[|*p* − *i*|]].

The rest of the proof is similar to the analysis of Theorem 5. □

**Theorem** **17.**  *In three-dimensional i.i.d. fast mobility networks, under the protocol model, the capacity can be expressed by:*

$$\lambda \left(n,m\right)=O\left(\sqrt[{3}]{\frac{D}{n^{2}}}\right)+\Theta \left(\frac{\sqrt[{3}]{m^{2}}W_{2}}{n}\right)$$

**Proof.**  Combining Theorems 15 and 16, we can prove the theorem above. □

### 6.4. Capacity of Three-Dimensional i.i.d. Slow Mobility Networks

Now we consider the three-dimensional i.i.d. slow mobility networks. Similar to the method used in network capacity analysis of two-dimensional i.i.d. slow mobility networks, we first calculate the bounds on the number of bits which are directly transmitted from source nodes to destination nodes without packet relaying. Furthermore, we analyze the number of bits which are delivered successfully from relay nodes to destination nodes.

**Theorem** **18.**  *In three-dimensional i.i.d. slow mobility networks, without packet relaying, the number of bits which are transmitted directly from source nodes to destination nodes satisfies:*

$$E\left[\Lambda^{d}[T]\right]\leq 6{\left(\frac{W_{1}T}{\Delta }\right)}^{\frac{3}{4}}\sqrt{n}$$

**Proof.**  Inequality Equation (16) in Theorem 7 is suitable for the three-dimensional i.i.d. slow mobility network. Besides, we have the following inequality which will be used in the following analysis:

(27) $$\sum_{B=1}^{\Lambda^{d}[T]}\sum_{h=1}^{H(B)}\frac{\Delta^{3}}{24}{\left(\alpha_{B}^{h}\right)}^{3}\leq \frac{W_{1}T}{\pi }$$

Hence, we have:

$$\begin{array}{ll} {\left(\sum_{B=1}^{\Lambda^{d}[T]}\sum_{h=1}^{H(B)}\alpha_{B}^{h}\right)}^{3} & \leq \left(\sum_{B=1}^{\Lambda^{d}[T]}\sum_{h=1}^{H(B)}1\right)\left(\sum_{B=1}^{\Lambda^{d}[T]}\sum_{h=1}^{H(B)}{\left(\alpha_{B}^{h}\right)}^{3}\right)\\ & \leq nW_{1}T\sum_{B=1}^{\Lambda^{d}[T]}\sum_{h=1}^{H(B)}{\left(\alpha_{B}^{h}\right)}^{3}\\ & \leq \frac{24nW_{1}^{2}T^{2}}{\Delta^{3}\pi } \end{array}$$

where the Cauchy-Schwarz inequality is used in the first inequality.

As $\sum_{h=1}^{H(B)}\alpha_{B}^{h}\geq L_{B}$, we obtain:

$$\sum_{B=1}^{\Lambda^{d}[T]}L_{B}\leq \sqrt[{3}]{\frac{24nW^{2}T^{2}}{\Delta^{3}\pi }}$$

Similar to the steps in Theorem 8, we have:

$$E\left[\Lambda^{d}[T]\right]-E\left[\sum_{B=1}^{\Lambda^{d}[T]}1_{L_{B}>L}\right]\leq \frac{4\pi }{3}nW_{1}TL^{3}$$

Therefore, we can conclude that:

$$\begin{array}{ll} \sqrt[{3}]{\frac{24nW^{2}T^{2}}{\Delta^{3}\pi }} & \geq E\left[\sum_{B=1}^{\Lambda^{d}[T]}L_{B}\right]\\ & \geq LE\left[\sum_{B=1}^{\Lambda^{d}[T]}1_{L_{B}>L}\right]\\ & \geq L\left(E\left[\Lambda^{d}[T]\right]-\frac{4\pi }{3}nW_{1}TL^{3}\right) \end{array}$$

Let $L=\sqrt[{3}]{\frac{E\left[\Lambda^{d}[T]\right]}{2\pi nW_{1}T}}$, we have Theorem 18. □

**Theorem** **19.**  *In three-dimensional i.i.d. slow mobility networks, if packet relaying is allowed, we have the following inequality:*

$$E\left[\Lambda^{r}[T]\right]\leq 6{\left(\frac{W_{1}T}{\Delta }\right)}^{\frac{3}{4}}\sqrt[{4}]{n^{2}D}$$

**Proof.**  Similar to the proof process, we have:

$$E\left[\sum_{B=1}^{\Lambda^{r}[T]}1_{L_{B}\leq L}\right]\leq \frac{4\pi }{3}nW_{1}TDL^{3}$$

Hence, we have the following inequalities:

$$\begin{array}{ll} E\left[\sum_{B=1}^{\Lambda^{r}[T]}L_{B}\right] & \geq E\left[\sum_{B=1}^{\Lambda^{r}[T]}L_{B}1_{L_{B}>L}\right]\\ & \geq E\left[\sum_{B=1}^{\Lambda^{r}[T]}1_{L_{B}>L}\right]L\\ & \geq \left(E\left[\Lambda^{r}[T]\right]-E\left[\sum_{B=1}^{\Lambda^{r}[T]}1_{L_{B}\leq L}\right]\right)L\\ & \geq E\left[\Lambda^{r}[T]\right]L-\frac{4\pi }{3}nW_{1}T\pi DL^{4} \end{array}$$

Based on the argument of Theorem 1, we have:

$$\sum_{B=1}^{\Lambda^{r}[T]}L_{B}\leq \sqrt[{3}]{\frac{24nW^{2}T^{2}}{\Delta^{3}\pi }}$$

Hence, we have:

$$\sqrt[{3}]{\frac{24nW^{2}T^{2}}{\Delta^{3}\pi }}\geq E\left[\Lambda^{r}[T]\right]L-\frac{4\pi }{3}nW_{1}T\pi DL^{4}$$

Let $L=\sqrt[{3}]{\frac{E\left[\Lambda^{r}[T]\right]}{2\pi nW_{1}T}}$, we have:

$$E\left[\Lambda^{r}[T]\right]\leq 6{\left(\frac{W_{1}T}{\Delta }\right)}^{\frac{3}{4}}\sqrt[{4}]{n^{2}D}$$

**Theorem** **20.**  *In three-dimensional i.i.d. slow mobility networks, the capacity contributed by ad hoc transmission mode is:*

$$\lambda_{A}\left(n\right)=O\left(\sqrt[{4}]{\frac{D}{n^{2}}}\right)$$

**Proof.**  Similar to the proof of Theorem 4, we have:

$$E\left[\Lambda [T]\right]\leq 6{\left(\frac{W_{1}T}{\Delta }\right)}^{\frac{3}{4}}\sqrt{n}\left(\sqrt[{4}]{D}+1\right)$$

Hence we have $E\left[\frac{\Lambda \left[T\right]}{n}\right]\leq O\left(\frac{\sqrt[{4}]{n^{2}D}}{n}\right)$. □

Theorem 16 implies that in three-dimensional networks, the capacity contributed by helping transmission mode is $\Theta \left(\sqrt[{3}]{m^{2}}W_{2}\right)$, hence we can obtain the throughput capacity of the whole networks:

**Theorem** **21.**  *In three-dimensional i.i.d slow mobility networks, under the protocol model, the capacity is:*

$$\lambda \left(n,m\right)=O\left(\sqrt[{4}]{\frac{D}{n^{2}}}\right)+\Theta \left(\frac{\sqrt[{3}]{m^{2}}W_{2}}{n}\right)$$

**Proof.**  Combining Theorems 16 and 20, we can prove the theorem above. □

We have obtained the capacities of the four types of heterogeneous mobile networks in this section. By analyzing these capacity expressions, we will get some interesting conclusions. The analysis is presented in the following section.

## 7. Capacity Analysis

**Figure 9:**
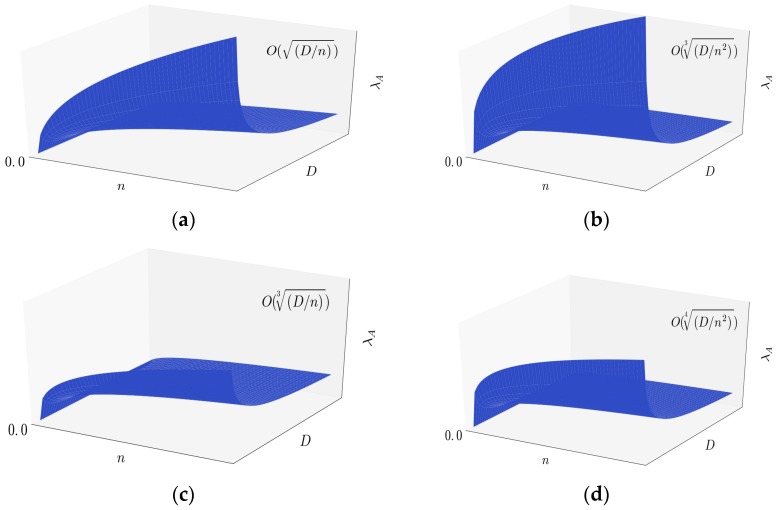
(**a**) *λ_A_* of two-dimensional i.i.d. fast mobility model; (**b**) *λ_A_* of three-dimensional i.i.d. fast mobility model; (**c**) *λ_A_* of two-dimensional i.i.d. slow mobility model; (**d**) *λ_A_* of three-dimensional i.i.d. slow mobility model.

In the Section above we have obtained the throughput capacity of the four types of mobile networks. In this section we analyze the impact of some important parameters such as *n*, *D* and *m* to the network capacity. Firstly we list the capacity contributed by *ad hoc* transmission mode as follows: in two-dimensional i.i.d. fast mobility model, we have $\lambda_{A}(n)=O\left(\sqrt{\frac{D}{n}}\right)$; in two-dimensional i.i.d. slow mobility model, we have $\lambda_{A}(n)=O\left(\sqrt[{3}]{\frac{D}{n}}\right)$; in three-dimensional i.i.d. fast mobility model, we have $\lambda_{A}(n)=O\left(\sqrt[{3}]{\frac{D}{n^{2}}}\right)$; and in three-dimensional i.i.d. slow mobility model, we have $\lambda_{A}(n)=O\left(\sqrt[{4}]{\frac{D}{n^{2}}}\right)$. Figure 9 shows *λ_A_*(*n*) of the four types of networks. 

**Figure 10:**
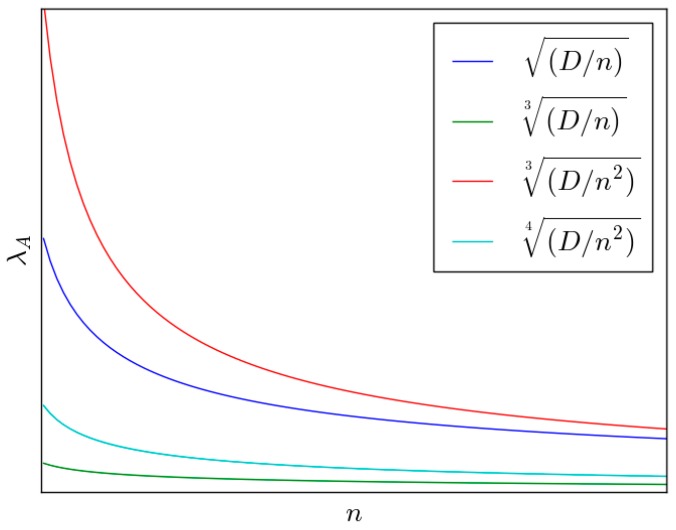
Relationship of *λ_A_*(*n*) and the number of normal nodes.

The relationship of *λ_A_*(*n*) and *n* is shown in Figure 10. We find that fast mobility networks have much higher capacity than the slow mobility networks, especially in the case that the number of mobile nodes is small. The reason is that in fast mobility networks, the source nodes have more opportunities to communicate with the destination nodes directly. If the number of mobile nodes is small, the fast mobility is one of the most important factors which influence the network capacity. As *n* goes to infinity, the number of mobile nodes offers many opportunities for source nodes to meet destination nodes, even in slow mobility networks. Hence, if the number of mobile nodes is large enough, the capacities of the four types of networks are similar.

Let *D* = Ω(*n*^2^), we have *λ_A_* = Θ(1). In this case, the four types of mobile networks have the same capacity, which is Θ(1). It means that in delay tolerant networks, the network capacity can achieve constant order, and the price is that the delay will go to infinity. Of course, the delay constraint *D* is approximated in the analysis above. Concretely, in two-dimensional mobile network model, *D* = Ω(*n*); in three-dimensional mobile network model, *D* = Ω(*n*^2^). However, since *n* is assumed to be large enough, *D* is not limited in both the two cases.

**Figure 11:**
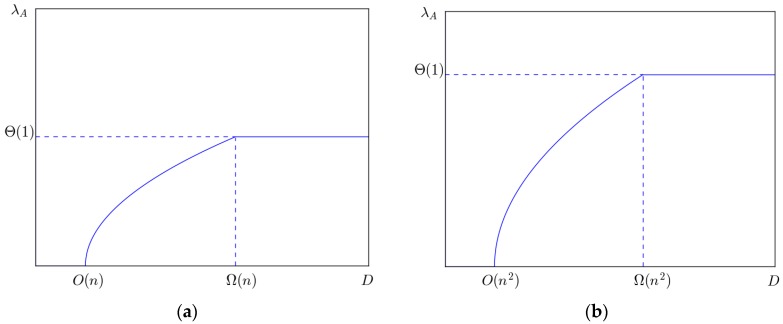
(**a**) The relationship of *λ_A_* and *D* in two-dimensional network model; (**b**) The relationship *λ_A_* of and *D* in three-dimensional network model.

Let *D* = *O*(*n*), we have *λ_A_* = 0. In this case, the capacity will decrease to zero as *n* goes to infinity. Concretely, in two-dimensional mobile network model, *D* = *O*(*n*); in three-dimensional network model, *D* = *O*(*n*^2^). It implies that if the delay is assumed to be very small, the number of bits transmitted by *ad hoc* transmission mode is very little. Our analysis is shown in Figure 11.

**Figure 12:**
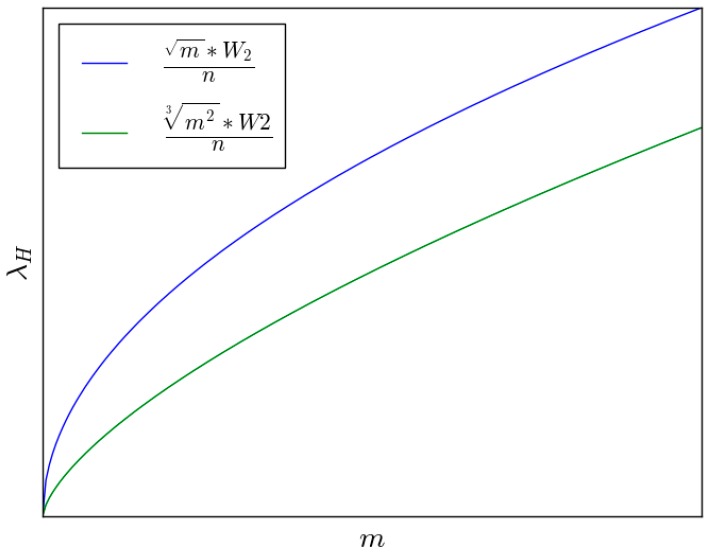
The relationship of between capacity and the number of helping nodes.

When *D* falls in between *O*(*n*) and Ω(*n*), there is a positive correlation between *λ_A_* and the delay constraint. Theorems 5 and 16 offer the throughput capacity of backbone networks. Figure 12 shows the relationship of throughput capacity and the number of helping nodes.

Combining the analysis above, we reach the following conclusions:

(1) In two-dimensional networks:
  $$\lambda \left(n,m\right)=\{\begin{array}{ll} \Theta \left(1\right)+\Theta \left(\frac{\sqrt{m}W_{2}}{n}\right), & \text{if}\;D=\Omega \left(n\right)\\ \Theta \left(\frac{\sqrt{m}W_{2}}{n}\right), & \text{if}\;D=O\left(n\right) \end{array}$$

If the delay is allowed to be larger than some fixed constant *D* = Ω(*n*), the capacity is $\Theta \left(1\right)+\Theta \left(\frac{\sqrt{m}W_{2}}{n}\right)$. In this condition the network capacity can achieve constant order. Specially, if *m* = Ω(*n*), most of packets can be delivered to the destination nodes with the help of backbone network. It implies that if the number of helping nodes is large enough, network capacity can stay constant order, and the delay can be limited, too. Similarly, if *m* = *o*(*n*), most of the packets will be transmitted by *ad hoc* transmission mode. It means that if the number of helping nodes is small, though the capacity is also constant order, the delay can not be ensured.

If the delay is limited (*D* = Ω(*n*)), the network capacity is $\Theta \left(\frac{\sqrt{m}W_{2}}{n}\right)$. In this condition, most of the packets will be transmitted by helping transmission mode. The number of helping nodes is the determining factor of the throughput capacity.

(2) In three-dimensional mobile networks:
  $$\lambda \left(n,m\right)=\{\begin{array}{ll} \Theta \left(1\right)+\Theta \left(\frac{\sqrt[{3}]{m^{2}}W_{2}}{n}\right), & \text{if}\;D=\Omega \left(n^{2}\right)\\ \Theta \left(\frac{\sqrt[{3}]{m^{2}}W_{2}}{n}\right), & \text{if}\;D=O\left(n^{2}\right) \end{array}$$

We can reach similar conclusions to two-dimensional mobile networks. According the analysis above, we can conclude that the number of helping nodes in the network is a key factor which affects the throughput capacity. If the number of helping nodes is large enough, the capacity can stay constant with the help of the backbone network. In this case, the time delay can be ensured. If the number of helping node is small, most of the packets have to be delivered in multi-hop fashion. In this case, though the throughput capacity can achieve constant order too, the delay will go to infinity. The delay constraint *D* is also an important factor on the network capacity. If *D* is not limited, the capacity contributed by *ad hoc* transmission mode can achieve Θ(1), which means that the network capacity of the whole network can achieve a constant order. If *D* is constrained, the capacity contributed by *ad hoc* mode is almost zero and most of packets have to be delivered by helping transmission mode.

## 8. Conclusions

In this paper we propose a new heterogeneous mobile network architecture, which is composed of normal nodes and more powerful helping nodes. Four types of mobile network model are investigated: two-dimensional i.i.d. fast mobility model, two-dimensional i.i.d. slow mobility model, three-dimensional i.i.d. fast mobility model and three-dimensional i.i.d. slow mobility model. First we provide a brief description of our network model. We analyze the throughput capacity of the four types of network model intuitively by virtual channel systems. Then we obtain the network capacity by strict mathematical proof respectively. We find that the throughput capacity is $O\left(\sqrt{\frac{D}{n}}\right)+\Theta \left(\frac{\sqrt{m}W_{2}}{n}\right)$ in two-dimensional i.i.d. fast mobility model, $O\left(\sqrt[{3}]{\frac{D}{n}}\right)+\Theta \left(\frac{\sqrt{m}W_{2}}{n}\right)$ in two-dimensional i.i.d. slow mobility model, $O\left(\sqrt[{3}]{\frac{D}{n^{2}}}\right)+\Theta \left(\frac{\sqrt[{3}]{m^{2}}W_{2}}{n}\right)$ in three-dimensional i.i.d. fast mobility model and $O\left(\sqrt[{4}]{\frac{D}{n^{2}}}\right)+\Theta \left(\frac{\sqrt[{3}]{m^{2}}W_{2}}{n}\right)$ in three-dimensional i.i.d. slow mobility model. Finally we analyze the asymptotic expressions we obtained. We consider two extreme cases: *D* is not limited and *D* is limited, where *D* is the delay constraint. We find that if *D* is not limited, the capacity contributed by *ad hoc* transmission mode can achieve constant order, while the price is that the delay will go to infinity. If *D* is limited, the capacity contributed by *ad hoc* transmission mode will decrease to zero. Hence there is a positive correlation between the throughput capacity and *D*. We also find that the number of helping nodes in the network is an important factor on the network capacity greatly. If the number of helping nodes is large enough, most of packets can be transmitted by helping transmission mode. In this condition the network capacity can achieve constant order without a long time delay. If the number of helping nodes is small, most of packets have to be transmitted by helping transmission mode, and the time delay can not be ensured.

## Figures and Tables

**Table 1:** Notations.

Notation	Definition
*W*	The total bandwidth of the networks, which means the highest transmission rate of each node.
*W*_1_	The bandwidth of *ad hoc* transmission mode.
*W*_2_	The bandwidth of uplink sub-channel in helping transmission mode.
*W*_3_	The bandwidth of downlink sub-channel in helping transmission mode.
*n*	The number of *ad hoc* nodes in the networks.
*m*	The number of helping nodes in the networks.
*D*	The delay constraints.
Δ	The guard zone size.
*L*_1_	The transmission radius of source nodes in virtual channel model.
*L*_2_	The transmission radius of relay nodes in virtual channel model.
Λ[*T*]	The total number of bits successfully delivered to destination nodes in *T* time slots.
*e_B_*	The node storing bit *B*.
*L_B_*	The minimum distance between *d_B_* and *e_B_*.
*λ_A_*(*n*)	The per-node throughput contributed by *ad hoc* transmission mode.
*λ_H_*(*n*)	The per-node throughput contributed by helping transmission mode.
*λ*(*n*)	The per-node throughput capacity of the network.
*H*(*B*)	The number of hops of bit *B* in slow mobility model.
$\alpha_{B}^{h}$	The transmission radius of bit *B* in its *h*th hop in slow mobility model.
